# Transcriptomic Analysis and Salt-Tolerance Gene Mining during Rice Germination

**DOI:** 10.3390/genes14081556

**Published:** 2023-07-29

**Authors:** Xiao Han, Zhihai Wu, Fangbiao Liu, Yu Wang, Xiaoshuang Wei, Ping Tian, Fenglou Ling

**Affiliations:** Faculty of Agronomy, Jilin Agricultural University, Changchun 130118, China; hanxiaoyy@jlau.edu.cn (X.H.); wuzhihai@jlau.edu.cn (Z.W.); liu310675119@163.com (F.L.); 17543559353@163.com (Y.W.); weixiaoshuang@jlau.edu.cn (X.W.); tianping@jlau.edu.cn (P.T.)

**Keywords:** rice, germination period, salt stress, transcriptome, differential expression

## Abstract

Salt stress is an important environmental factor affecting crop growth and development. One of the important ways to improve the salt tolerance of rice is to identify new salt-tolerance genes, reveal possible mechanisms, and apply them to the creation of new germplasm and the breeding of new varieties. In this study, the salt-sensitive japonica variety Tong 35 (T35) and salt-tolerant japonica variety Ji Nongda 709 (JND709) were used. Salt stress treatment with a 150 mmol/L NaCl solution (the control group was tested without salt stress treatment simultaneously) was continued until the test material was collected after the rice germination period. Twelve cDNA libraries were constructed, and 5 comparator groups were established for transcriptome sequencing. On average, 9.57G of raw sequencing data were generated per sample, with alignment to the reference genome above 96.88% and alignment to guanine-cytosine (GC) content above 53.86%. A total of 16,829 differentially expressed genes were present in the five comparison groups, of which 2390 genes were specifically expressed in T35 (category 1), 3306 genes were specifically expressed in JND709 (category 2), and 1708 genes were differentially expressed in both breeds (category 3). Differentially expressed genes were subjected to gene ontology (GO), functional enrichment analysis, and Kyoto Encyclopedia of Genes and Genomes (KEGG) pathway analysis, which revealed that these genes belonged to three main classes: molecular function, cellular components, and biological processes. KEGG pathway analysis showed that the significantly enriched pathways for these differentially expressed genes included phenylpropane biosynthesis, phytohormone signaling, and the interaction of plants with pathogens. In this study, we provided a reference for studying the molecular mechanism underlying salt tolerance during germination.

## 1. Introduction

Rice (*Oryza sativa* L.) is one of the major global food crops. It occupies an extremely important position in food production and food consumption. Salt stress is an important environmental factor that leads to reduced rice production, and severe stress can cause plant death and affect the stable production of rice [1,2]. The existing saline–alkali land area in the world is approximately 9.6 × 10^9^ hm^2^ [3], and the salt damage area accounts for approximately 1/5 of the arable area [4]. The reasonable implementation of soil and water management technology and the application of chemical amendments can alleviate the damage caused by salt on rice within a certain range; however, this method is costly and slow in effect and cannot be used on a large scale [4]. Therefore, to address the issue of rice persecution at its core, research should be conducted on the salt tolerance of rice, enhancing salt tolerance and developing salt-tolerant rice varieties, which is an effective means of ensuring food security and improving the ecological environment in saline–alkali areas of the worldwide [1,2].

Under salt stress, rice plants initially show various hindered growth and developmental processes [5]. These include resistance to osmotic metabolism, strengthening ion toxicity, and the reinforcement of abiotic stress. The physiological regulation mechanism of plant salt tolerance primarily involves the regulation of osmotic metabolism and nutrient transport, resistance regulation, and hormone regulation [6]. Changes in the external environment can cause changes in metabolites in plants, and to accumulate substantial metabolic production in plant cells, various metabolism-related products are significantly elevated to regulate osmotic balance when subjected to salt stress [7]. Plant endogenous hormones also play an important regulatory role under salt stress conditions and can improve the activity of related protective enzymes by inducing the relevant defense system or enhancing the resistance to alleviate salt damage and relieve the salt stress by improving the ion absorption and distribution of cells [8,9,10,11,12]. Plants are different from animals; to resist abiotic and biotic stresses derived from the environment, they have developed a fine, efficient, and complete defense system during the long evolutionary process [13], and many protective enzymes play important roles in stress resistance in plants [14].

Exploring the tolerance mechanism of salt stress, morphological and physiological changes and differences in the expression of genes related to salt tolerance is of great theoretical and practical significance for further cultivating salt-tolerant rice varieties and guiding rice production in saline–alkali land. With the development of modern biotechnology and genetic engineering technologies, genes increasingly associated with salt tolerance in rice have been mined and validated, accelerating the process of improving rice salt-tolerant germplasm and bringing new progress to rice salt-tolerant breeding [15]. Therefore, identifying more genes related to salt tolerance has become the focus of current rice salt tolerance breeding efforts. In this study, salt-sensitive and salt-tolerant rice plants were subjected to salt stress during the germination stage. Transcriptome sequencing technology (RNA-Seq) was used to analyze the differentially expressed genes (DEGs) in the rice germination stage under different genotypes and salt stress treatments, and the related genes were mined. The analysis of differentially expressed genes in the germination stage under salt stress can accelerate the mining and identification of new genes related to salt tolerance during the germination stage, which provides an important theoretical basis for germplasm innovation and new varieties of salt-tolerant rice.

## 2. Results

### 2.1. Phenotypic Differences between T35 and JND709 under Salt Stress

**Figure 1:**
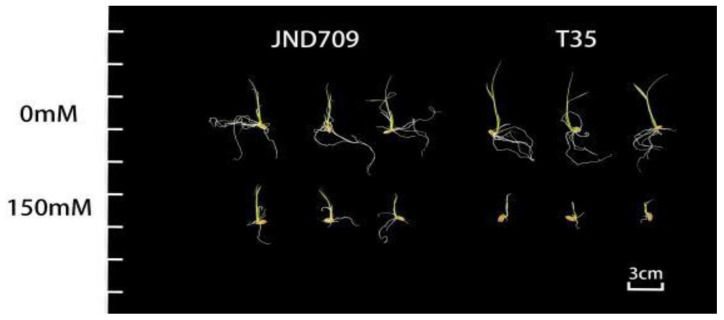
Germination of T35 variety and JND709 variety under different salt concentration treatment conditions.

There was no significant difference in the germination status of the T35 and JND709 seeds under normal culture conditions. The growth of both T35 and JND709 was significantly inhibited after salt stress treatment; T35 was inhibited to a greater extent than JND709, and the bud length was significantly shorter. These results indicate that T35 was more sensitive to salt than JND709 (Figure 1).

### 2.2. Overall Evaluation of the RNA-Seq Data

Twelve cDNA libraries were constructed from T35 and JND709 samples under the two treatments, each with three replicates. Raw sequencing data (Raw data) were obtained for 117G, which produced 9.57G of raw sequencing data per sample and 94.383G of clean data (Supplementary Table S1). As shown in Table 2, the Q20 base percentage was above 97.99%, Q30 base percentage was above 93.5%, and GC content was above 53.86%. A higher alignment ratio for all samples in the genome can satisfy the need for the classification of differentially expressed genes. After quality control, the sequence was aligned with the reference genome using HISAT2 and aligned with the results obtained using RSeQC statistics. Using Nipponbare (LOC_Os09g36350) as the reference genome for reference analysis, twelve samples had a pair ratio above 96.88%, and the two-end alignment rate ranged from 90.99% to 93.32% (Supplementary Table S2), further illustrating the high accuracy of the sequencing data. 

### 2.3. Sample Relationship Analysis

**Figure 2:**
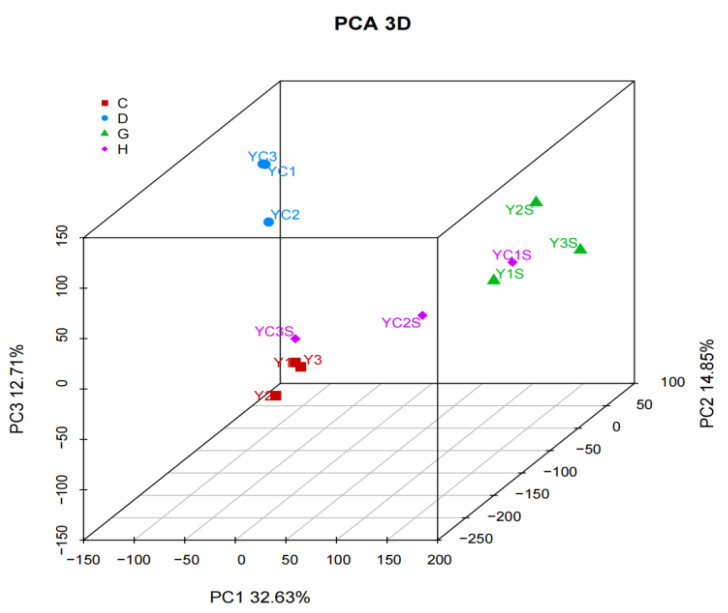
PCA between all samples. C: JND709 varietiy treated with 150 mmol/L of a NaCl solution; D:T35 variety treated with 150 mmol/L of NaCl; G: JND709 variety treated with distilled water; H: T35 variety treated with distilled water. Numbers 1, 2, and 3 represent the three replicates of the sample in this figure and below.

**Figure 3:**
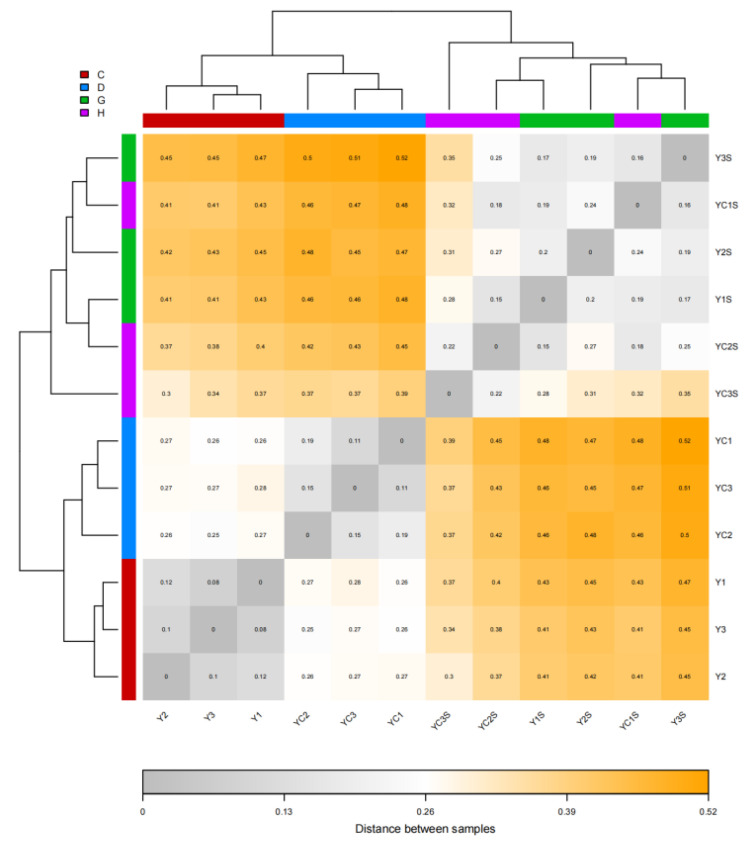
Correlation heatmap of sequencing samples.

Principal component analysis between samples is shown in Figure 2. The long distance between samples and salt stress indicated that salt stress caused significant differences in the gene expression levels of each sample. At the same time, JND709 salt-stressed and unstressed samples clustered together with small differences in gene expression levels. However, the distance between the T35 salt-stressed and unstressed samples and the differences in the gene expression levels indicated that the expression response of T35 was low after salt stress. Based on the correlation heat map of sequencing samples (Figure 3), showing a high correlation coefficient between sequencing samples with the same treatment, there were large differences in sample gene expression levels between different treatments and varieties, and the correlation coefficient was small. This indicates that high repetition between sequencing samples resulted in less systematic error, and the results were highly credible.

Figure 2 shows the PCA of all samples: C: JND709 treated with 150 mmol/L of a NaCl solution; D: T35 treated with 150 mmol/L of a NaCl solution; G: JND709 treated with distilled water; H: T35 treated with distilled water. Numbers 1, 2, and 3 represent the three replicates of the sample in Figure 2 and below.

### 2.4. Analysis of Differentially Expressed Genes

**Figure 4:**
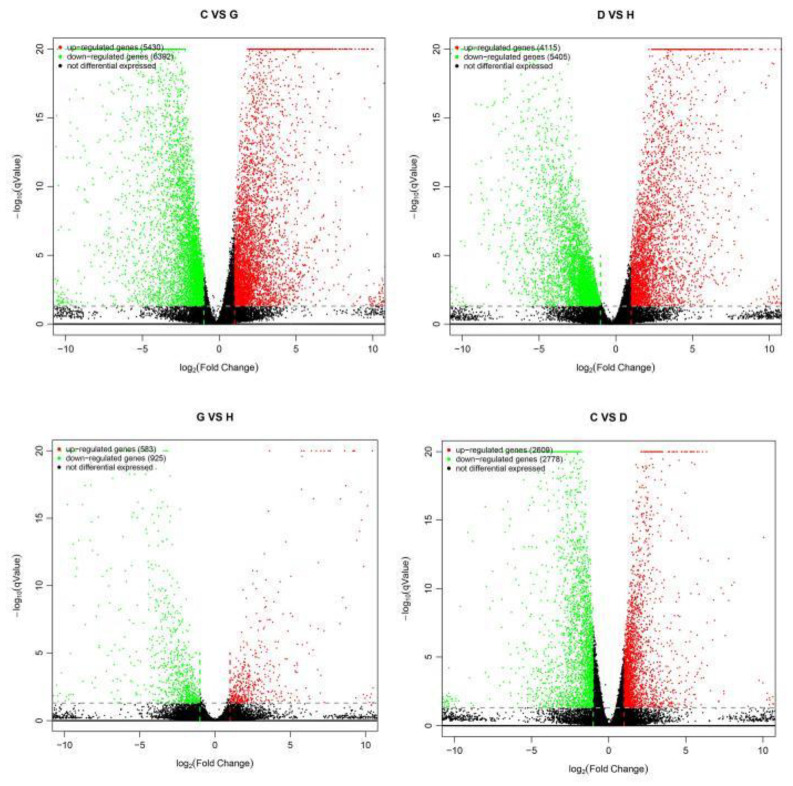
Volcano map of differentially expressed genes between different salt stress treatments.

The number of differentially expressed genes in each comparison group is shown in Figure 4 and Table 1. The salt-sensitive material T35 had 9520 differentially expressed genes before and after salt stress, of which 4115 were upregulated and 5405 were downregulated. JND709 had 11,822 differentially expressed genes before and after salt stress, with 5430 upregulated and 6392 downregulated genes. After salt stress, there were 5387 differentially expressed genes: 2609 genes were upregulated, and 2778 genes were downregulated.

**Figure 5:**
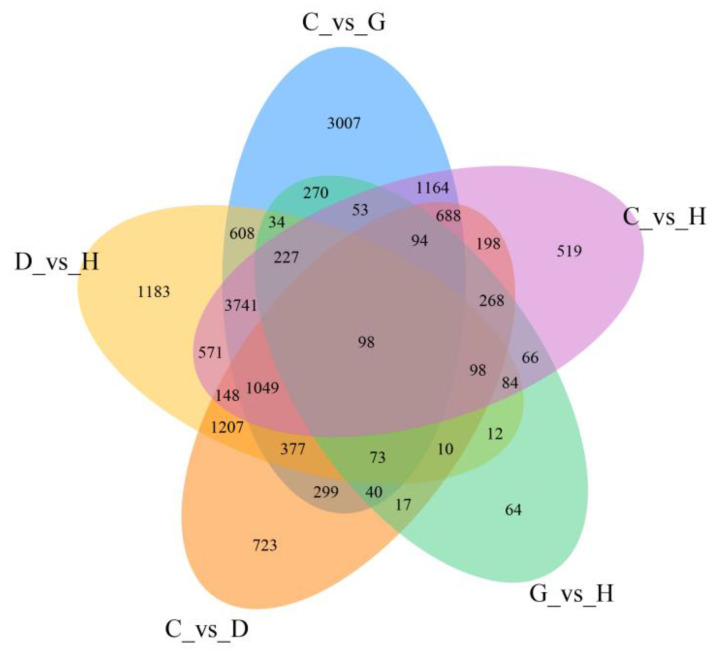
Venn diagram of differentially expressed genes under different treatments.

By comparing the differentially expressed genes of the same rice variety in different environments and different rice varieties in the same environment, five comparison groups were obtained, and 16,829 differentially expressed genes were identified in the five comparison groups. Differentially expressed genes were divided into 30 subgroups (Figure 5). Excluding genes unrelated to G vs. H to salt stress, the remaining 15 subgroups were divided into three categories: genes specifically expressed by T35 (category 1), genes specifically expressed by JND709 (category 2), and genes differentially expressed in both varieties (category 3). Of these, 2390 were available for category 1, 3306 were available for category 2, and 1708 were available for category 3 (Table 2).

With|log2 (fold change)| ≥ 2 as the screening condition, a set of data for a comparison group must meet the condition FPKM ≥ 2. Genes from the three categories were screened separately, yielding 873 major active genes related to salt tolerance, including 219 from category 1, 284 from category 2, and 370 from category 3.

### 2.5. GO Term Enrichment Analysis Based on DEGs

**Figure 6:**
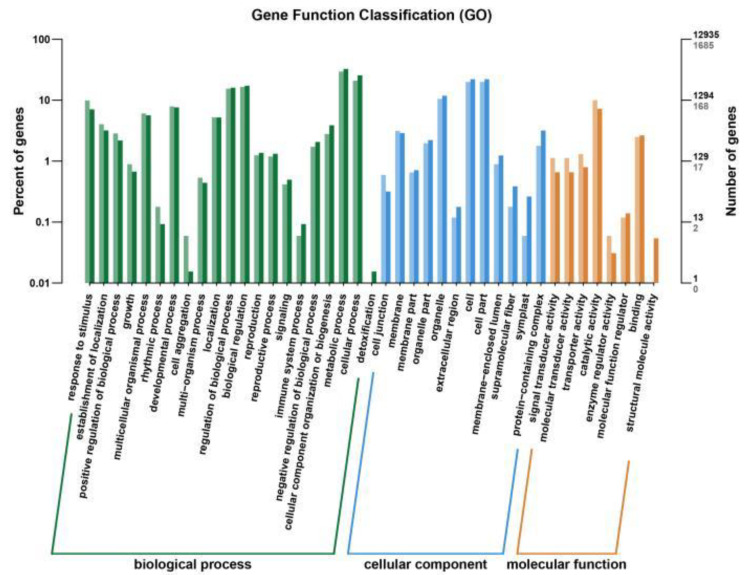
Functional annotation of differentially expressed genes: GO of JND709 and T35.

The GO functional annotation of the differentially expressed genes in JND709 and T35 under salt stress (Figure 6) revealed that 274 differentially expressed genes were classified into molecular functions, 1007 differentially expressed genes were classified into cellular components, and 2145 differentially expressed genes were classified into biological processes. Differential genes in molecular functions are mainly involved in catalytic activity (169), binding (42), and signal transduction activity (22). Differentially expressed genes in cellular components were mainly concentrated in cells (336), cellular components (336), and organelles (177). Differentially expressed genes in biological processes were mainly concentrated in metabolic processes (496), cellular processes (352), and biological regulation (278).

### 2.6. KEGG Pathway Analysis

**Figure 7:**
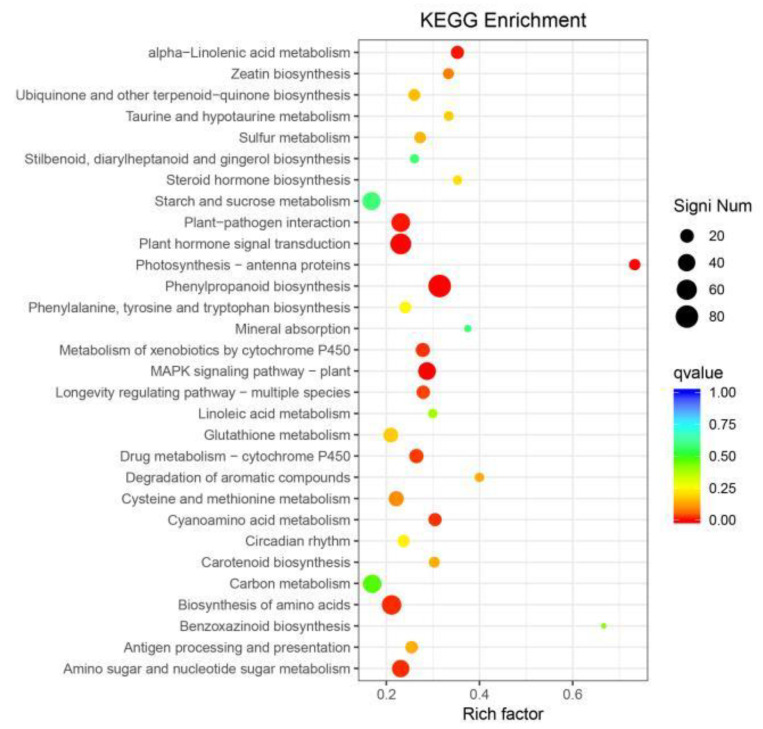
KEGG enrichment analysis of differentially expressed genes in JND705 and T35.

To accurately analyze the changes occurring in in vivo metabolic pathways in response to salt stress during juvenile development, the KEGG classification statistical analysis of the targeted differential genes revealed that 819 genes were annotated, involving 232 pathways (Figure 7). The metabolic pathways significantly enriched for these differentially expressed genes mainly included α-Linolenic acid metabolism, for which the most significant performance was by 12-oxo-phytodienoate reductase (LOC_Os06g11240); plant hormone signal transduction, for which the most significant performance was by SCP (LOC_Os07g03620); phenylpropanoid biosynthesis, for which the most significant performance was by dihydroflavonol-4-reductase (LOC_Os02g56690); the biosynthesis of amino acids, for which the most significant performances were by acetolactate synthase 1 (LOC_Os06g51280) and glutamine synthetase (LOC_Os03g12290); carbon metabolism, for which the most significant performance was by pyruvate phosphate dual kinase (LOC_Os03g31750); amino sugar and nucleotide sugar metabolism, for which the most significant performance was by chitinase (LOC_Os06g51050); starch and sucrose metabolism, for which the most significant performance was by pectinesterase (LOC_Os11g07090); and plant-pathogen interaction, for which the most significant performances were by SCP (LOC_Os07g03620) and calmodulin (LOC_Os12g12730). Twelve pathways showed significant enrichment (Q ≤ 0.05) (Table 3). Among them, 80 differentially expressed genes were enriched in phenylpropane biosynthesis pathways, 65 were enriched in phytohormone signal transduction pathways, and 49 were enriched in plant-pathogen interaction pathways. This suggests that these pathways play crucial roles in the plant response to salt stress.

### 2.7. qRT-PCR Validation of Transcriptome Sequencing Data

**Figure 8:**
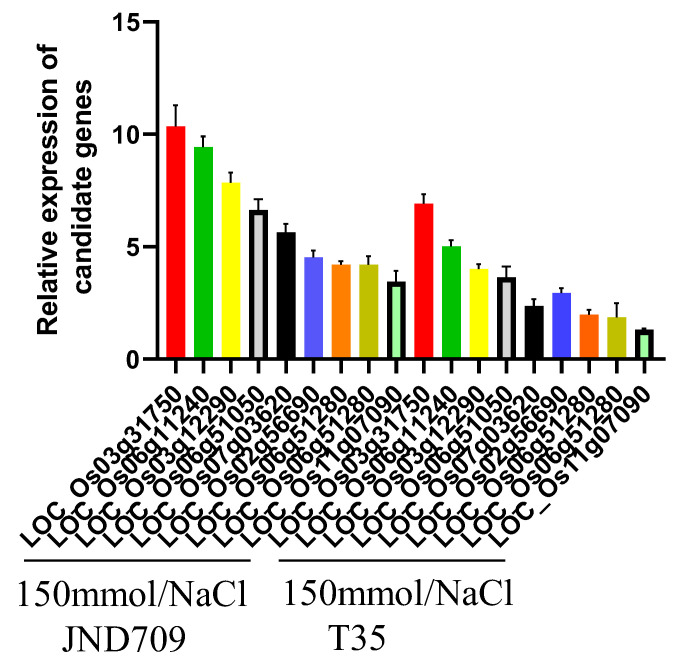
Comparison of differentially expressed gene transcriptome and qRT-PCR results.

To verify the authenticity of the transcriptome sequencing results, eight differentially expressed genes were selected from the salt stress-related genes, and their transcript levels were verified by qRT-PCR in test tissues treated with T35 and JND709 salt stress. The transcriptome sequencing data were consistent with the trends of the differentially expressed genes obtained by qRT-PCR validation (Figure 8), indicating that the transcriptome sequencing data were more reliable.

## 3. Discussion

The regulatory mechanisms in plants in response to stress are complex. When cells experience salt stress, they transmit the signal to the corresponding receptors, which then control the related gene expression and physiological response while responding to the level of transcription and translation [16]. Little research has been conducted on the mechanisms underlying salt tolerance during rice germination under salt stress conditions. Therefore, the transcriptome-level sequencing analysis of rice germination materials under salt stress can rapidly and accurately identify the mechanisms of salt tolerance during the germination stage. In this study, sequencing before and after the rice germination period of salt-tolerant material JND709 and salt-intolerant material T35 showed that all indicators met the requirements, indicating that the overall sequencing quality was good.

When rice is in an unfavorable environment during growth and development, it often activates related genes and metabolic pathways to reduce the damage caused by various stresses. Therefore, the metabolites and nutritional qualities of rice grains are highly susceptible to change. Phenylaprapanoid metabolism is one of the most important secondary metabolic pathways in plants. The initiation reaction is catalyzed by phenylalanine ammonia lyase, cinnamate 4_hydroxylase, and 4-Coumarate: coenzyme A ligase, which then produces lignin. Flavonoids are the most important branched metabolites [17]. Flavonoids play an important role as antioxidants in plant abiotic stress responses, and their growth and integrity can be controlled by regulating ROS homeostasis under stress [18]. Lignin is involved in the resistance to abiotic stress, and the upregulation of lignin biosynthesis genes leads to lignin deposition, the thickening of secondary cell walls, and enhanced plant stress resistance [19,20,21]. There are reports of the systematic analysis of metabolome changes in maize seedlings under neutral and alkaline salt stress using gas chromatography–tandem mass spectrometry (GC-MS). Alkaline salts change plant metabolism and inhibit photosynthesis, nitrogen metabolism, glycolysis, and the synthesis of various amino acids [22]. In this study, after salt stress, dihydroflavonol-4-reductase (LOC_Os02g56690) was upregulated in seedlings during JND709 germination and downregulated in T35. Therefore, it is speculated that phenylpropane plays a role in the young spike response to salt stress and may participate in the activation of related metabolic pathway genes. Few studies have reported the effects of salt stress on secondary metabolism in rice. The effects of salt stress on phenylpropane metabolic pathways in rice, the formation of the metabolite lignin and flavonoids, and the physiological basis of how these two metabolites are involved in regulating salt tolerance in rice are also not clear. To this end, the aim of this study was to use selected salt-sensitive rice varieties and salt-tolerant rice varieties as materials to study the relationship between the key enzymes and metabolites of phenylaprapanoid metabolism and their fertility indicators and to further analyze the physiological mechanism of salt tolerance via phenylaprapanoid metabolism to provide a theoretical basis and technical guidance for enriching the rice salt tolerance mechanism and variety breeding.

During plant growth, almost all life activities are regulated by hormones, and their response to salt stress is no exception. There is no obvious specificity for plant hormones. Usually, one hormone can regulate multiple physiological processes, and multiple hormones play roles in regulating the same physiological process [23]. Studies have shown that three main endogenous hormones are involved in the regulation of salt stress in rice. The first is salicylic acid (SA). Under salt stress conditions, salicylic acid can alleviate membrane lipid peroxidation damage by inducing antioxidant defense systems or enhancing antioxidant capacity and improving cell metabolism by reducing plasma membrane permeability, regulating ion absorption and distribution, and relieving the pressure of salt stress [24,25]. The second type is abscisic acid (ABA). ABA can induce a massive accumulation of the plant osmoregulator proline. To alleviate the osmotic stress and ion toxicity caused by excessive salinity, water balance is maintained [26], and the activity of related protective enzymes is improved [27], thereby alleviating plant salt damage. The third type is jasmonic acid (JA). This endogenous hormone is similar to ABA [28] and is also a defense-signaling molecule. It regulates stomatal closure by signal transduction to weaken transpiration and reduce water loss [29]. The expression of serine carboxypeptidase-like protein family genes is not only hormone-regulated but also stress-induced. The serine carboxypeptidase-like protein family has been shown to play crucial roles in plant growth and development in response to abiotic stresses. It is an important gene involved in ABA signaling that regulates rice grain filling and dormancy processes [30]. Additional studies have shown that serine carbohydrates in rice may be associated with the oxidative stress defense response caused by biotic or abiotic stress [31]. The serine carboxypeptidase gene in maize shows a trend of induced expression under both biotic and abiotic stress [32]. In this study, phytohormone-regulated genes, such as SCP (LOC_Os07g03620), were upregulated in JND709 germinated seedlings and downregulated in T35. It is speculated that plant hormones can promote the elongation of seedling cells during the JND709 germination stage, ensure the normal development of young ears, and improve tolerance to salt stress conditions. Currently, the resolution of salt-tolerant protease structures comes mostly from experiments using limited data. Subsequent experiments can use bioinformatics software to predict the amino acid composition characteristics, secondary structure, hydrophobic affinity, surface accessibility, conserved domains, tertiary structure, and key amino acids of the catalytic site of salt-serine-resistant proteases. This study aimed to provide a theoretical basis for an in-depth exploration of the salt tolerance mechanisms of serine proteases.

Plants face three major challenges in their life cycle: growth, defense, and reproduction. Growth and defense are the two challenges during the seedling stage before entering the reproductive stage. Generally, when plants face environmental stress, their growth is inhibited to varying extents. This may be related to the inhibition of photosynthesis and the transfer of more resources into the synthesis of defensive substances rather than growth. As a serious abiotic stress, salt damage can significantly weaken the disease resistance of rice, which may be closely related to a reduction in the viability of related protective enzymes. SOD is the most important active-oxygen-species-scavenging enzyme, which is closely related to plant resistance to stress (such as drought, salt damage, etc.) [33] and is also closely related to plant disease resistance. PPO is closely associated with plant disease resistance. This enzyme plays a disease resistance role by catalyzing the synthesis of polyphenols and lignin [34]. Under salt stress, large amounts of reactive oxygen species are produced in rice plants compared with those in the normal environment. Thus, membrane lipid peroxidation severely destroys the biological function of the cell membrane and can lead to cell death in severe cases [35]. Studies have indicated that salt-tolerant rice has a stronger ROS-scavenging capacity than salt-sensitive rice. SOD and POD activities in rice leaves first increased and then decreased with an increase in salt stress and showed a positive correlation within a certain range of salt concentrations [36,37]. The overexpression of the Nt GST/GPX gene in tobacco is effective in increasing the resistance of tobacco leaves to salt-damage environments [38]. In this study, the analysis of target genes after salt stress revealed that more genes related to plant antioxidants under salt stress conditions were upregulated in JND709 germinated seedlings and downregulated in T35. It is speculated that the strong salt tolerance of JND709 is due to the differential expression of these antioxidant enzymes between the two varieties. The regulation of the antioxidant system can effectively remove reactive oxygen species harmful to rice, protect the rice membrane system, maintain the normal membrane structure and function, and enhance tolerance in the salt-stress environment.

In stress biology studies, the focus of research has always been on calcium signals in plants. Calcium signals play a significant role in regulating plant stress responses as well as growth and developmental processes [39]. When plants are stressed by an adverse environment, kinases downstream of calcium ions, such as calcium-dependent protein kinases, calmodulins, and calmodulin-like proteins, are induced. In this study, we found that, among the target genes after salt stress, one calmodulin gene (LOC_Os12g12730) was upregulated in JND709 germinated seedlings and downregulated in T35 seedlings. Therefore, it is speculated that calcium signaling occupies a certain place in the young spike in response to salt stress and may be involved in the activation of various detoxification-related genes within cells.

## 4. Materials and Methods

### 4.1. Plant Materials and Treatments

The plant materials were the salt-sensitive japonica rice variety T35 and the salt-tolerant japonica rice variety JND709 provided by the Rice Research Institute of Jilin Agricultural University.

### 4.2. Salt Stress Treatment and Collection of Rice Seed Samples

We selected 600 healthy and intact T35 and JND709 seeds. First, we performed surface disinfection by soaking the seeds in 75% ethanol for 20 min. They were then soaked in a 10% sodium hypochlorite solution for 30 min for deep disinfection, and 50 seeds were randomly selected for a set of test materials. All groups of test materials were spread evenly in a 9 cm diameter Petri dish with two layers of filter paper.

For salt stress, the test groups were treated with 150 mmol/L of a NaCl solution, and the control group was treated with the same volume of distilled water. Three biological replicates were used for both the test and control groups. The JND709 150 mmol/L salt stress treatment was set as group C, the JND709 distilled water treatment was set as group G, the T35 150 mmol/L salt stress treatment was set as group D, and the T35 distilled water treatment was set as group H. All test materials were placed in the same artificial climate box for cultivation. The day temperature was set to 27.0 ± 0.5 °C, the night temperature was set to 25.0 ± 0.5 °C, and daily cultures lasted 14 h in the light and 10 h in the dark. All test materials were collected on day 10, flash-frozen in liquid nitrogen, and stored at −80 °C (Entrusted Bioengineering (Shanghai) Co., Ltd.). To complete RNA extraction, quality control, library construction, and transcriptome sequencing work, three replicates were performed for each test treatment sample.

### 4.3. Transcriptomic Analysis of the Salt-Tolerant Rice Varieties

Total RNA was extracted from rice samples treated with different salt conditions using a Tissue Total RNA Isolation Kit (Biological Bioengineering, Inc.). The five comparative rents were recorded as D vs. H, C vs. G, G vs. H, C vs. D, and C vs. H. cDNA libraries were sequenced using the Illumina Sequencing platform. Nipponbare was used as the reference genome for reference analysis. Raw sequencing data were assessed for quality using FastQC, and valid data for the sample reads were mixed by splicing using Trinity to obtain a unique sequence for each piece of information. Exon million mapped fragments per kilobase (FPKM) were calculated using HISAT2 software to assess the expressed genes. For samples with biological replicates, DESeq2 was used to identify the differentially expressed genes (DEGs). Significant differential gene screening conditions were set as follows: fold change ≥ 2 or ≤0.5 and *p* < 0.05. After the differentially expressed genes were selected, we performed differential GO classification of genes and KEGG metabolic pathway enrichment analysis. We further studied the distribution of differentially expressed genes in annotated functions and clarified the embodiment of sample differences in gene functions.

### 4.4. Verification by Quantitative Real-Time PCR

This test sample RNA was reverse transcribed using the Takara reverse transcription kit. The qRT-PCR reaction system configuration used was as follows: cDNA 1 μL, PRemix Ex Taq TMⅡ 10 μL, dd H2O 7 μL, Rox RefeRence Dye 0.4 μL, 10 μmol/L, and 0.8 μL each of the upper and lower primers. The reaction conditions were as follows: 95 °C for 1 min, 95 °C 15 s, 55 °C 30 s, 72 °C 60 s, 65 °C 5 s, and 95 °C 5 s with 40 cycles. Three biological and three technical replicates were used for each gene. Actin was used as a reference gene. Relative expression was calculated using 2^−∆∆Ct^ [40,41], and the primers used in this study are shown in Supplementary Table S3.

## 5. Conclusions

In this study, The salt-tolerant rice varietie JND709 and salt-intolerant rice varietie T35 were stressed by 150 mmol/L of NaCl. The germination materials were sequenced using RNA-seq. By comparing the Venn diagram of upregulated and downregulated genes under different varieties and treatments, 3426 differentially expressed genes were found to be upregulated in JND709 germinated seedlings and downregulated in T35 seedlings. These target genes were analyzed using GO function enrichment analysis and KEGG metabolic pathway enrichment analysis to elucidate the differential gene enrichment pathways and molecular functions. Differentially expressed genes were classified into molecular functions, cellular components, and biological processes, which significantly or very significantly affected 12 metabolic pathways. Among them, the differential expression differences in the phenylpropane biosynthesis pathway, the phytohormone pathway, and the plant-pathogen interaction pathway are the most prominent. This indicates that amino acid metabolism and secondary metabolism are significantly enhanced and may be the key genes regulating salt tolerance. These results indicate that rice will undergo complex transcriptional regulatory network changes in response to salt stress, which regulates the whole resistance (sensory) salt process via signal transmission between signaling pathways. These may be the primary factors contributing to the relative salt tolerance of salt-tolerant varieties.

## Figures and Tables

**Table 1:** Statistics of differentially expressed genes.

Compare Type	Upregulated	Downregulated	Number
C vs. G	5430	6392	11,822
D vs. H	4115	5405	9520
C vs. D	2609	2778	5387
G vs. H	583	925	1508

**Table 2:** Classification of three categories of DEGs.

Categories	Subgroups	Number of DEGs
1	Only D vs. H	1183
	D vs. H, D vs. C	1207
2	Only C vs. G	3007
	C vs. G, D vs. C	299
3	Only D vs. C	723
	D vs. H, C vs. G	608
	D vs. H, C vs. G, D vs. C	377

**Table 3:** Data table of significant enrichment of pathways for each group.

ID of the KEGG	Description	Significant	Annotated	Qvalue
ko00940	Phenylpropanoid biosynthesis	80/819	254/6009	9.84 × 10^−12^
ko00196	Photosynthesis—antenna proteins	11/819	15/6009	2.50 × 10^−5^
ko04016	MAPK signaling pathway—plant	42/819	146/6009	7.11 × 10^−5^
ko04075	Plant hormone signal transduction	65/819	281/6009	0.000365623
ko00592	Alpha-Linolenic acid metabolism	18/819	51/6009	0.003193511
ko04626	Plant-pathogen interaction	49/819	212/6009	0.003462975
ko00520	Amino sugar and nucleotide sugar metabolism	40/819	173/6009	0.011626162

## Data Availability

Not applicable.

## Supplementary Materials

The following supporting information can be downloaded at: https://www.mdpi.com/article/10.3390/genes14081556/s1, Table S1: Sequencing data quality assessment sheet; Table S2: Comparative analysis with reference group genes; Table S3: The sequences for qRT-PCR primers.
