# Supplementary material for: Transcriptomic Analysis and Salt-Tolerance Gene Mining during Rice Germination
Source: Genes (Basel). 2023 Jul 29;14(8):1556. doi: 10.3390/genes14081556 (PMC10454240; doi:10.3390/genes14081556)
Supplement: Supplementary file 1 [file genes-14-01556-s001.zip › genes-2469419-supplementary.pdf]

Supplementary table S1. Sequencing data quality assessment sheet

	Total Reads Count(#)	Total Bases Count(bp)	AveRage Read Length(bp)	Q20 Bases Ratio(%)	Q30 Bases Ratio(%)	GC Bases Ratio(%)
C1	55565222	8065051109	145.15	98.18%	93.97%	57.00%
C2	40487402	5829561627	143.98	98.17%	94.00%	56.92%
C3	53184668	7459813603	140.26	98.03%	93.67%	56.66%
D1	42792912	6016334413	140.59	97.99%	93.50%	56.10%
D2	51971658	7379748459	142.00	98.22%	94.11%	57.03%
D3	40513120	5670996973	139.98	98.05%	93.68%	55.47%
G1	64956848	9318361091	144.54	98.45%	94.80%	55.03%
G2	66928786	9562734973	144.82	98.29%	94.35%	54.73%
G3	64666734	9348823210	144.98	98.31%	94.40%	53.86%
H1	60330160	8720192072	143.45	98.26%	94.24%	54.30%
H2	57120578	8271953177	142.88	98.33%	94.47%	54.26%
H3	60283556	8739760411	144.57	98.45%	94.76%	54.28%

Supplementary table S2. CompaRative analysis with RefeRence gRoup genes

	Total Reads	Total mapped	Reads mapped in pRopeR paiRs
C1	54584920(100.00%)	53285847(97.62%)	50937090(93.32%)
C2	39620080(100.00%)	38497648(97.17%)	36624878(92.44%)
C3	50294160(100.00%)	48724162(96.88%)	45761786(90.99%)
D1	41921958(100.00%)	41067198(97.96%)	39108756(93.29%)
D2	51193846(100.00%)	50156305(97.97%)	47635818(93.05%)
D3	39942868(100.00%)	38995210(97.63%)	37018374(92.68%)
G1	64416044(100.00%)	62657908(97.27%)	58706198(91.14%)
G2	66377324(100.00%)	64457041(97.11%)	60538158(91.20%)
G3	63766140(100.00%)	62421929(97.89%)	58689166(92.04%)
H1	58599184(100.00%)	57062062(97.38%)	53595212(91.46%)
H2	56738904(100.00%)	55504709(97.82%)	52630736(92.76%)
H3	59691570(100.00%)	58439259(97.90%)	55362832(92.75%)

Supplementary table S3. The sequences foR qRT-PCR pRimeRs

Gene ID	F-pRimeR（5'—3'）	R-pRimeR（5'—3'）
LOC_Os06g11240	GGTGATAGCCGATGGATATGCT	GTCCGGATTGGCCAAGAA
LOC_Os07g03620	CGCGTCGCCTGCAATAC	TTACCACGCGGGAAGTAGTTG
LOC_Os02g56690	AACTTTTCCCACAGTACCCCG	AGCCCAAATCCTTGAGCCTC
LOC_Os06g51280	GGATGCATGGCACCGTGTA	CACGCCGAACGCAAGAA
LOC_Os03g12290	GTTGGAGGATCGGGCATAGACCTC	CACTTGGTCAGCAGCGGCGATGCCAA
LOC_Os03g31750	CGCTTCGCCTACGACTCCTA	GAATGCGGTATGTCCATAACGA
LOC_Os06g51050	GCGAGATCACCAACATCATCA	CAATCCAGGTTATCGCCATAGC
LOC_Os11g07090	GGCCTCATGTCCTCTGCTTTC	CAGATCAGCGGCCGAGAT
